# The tree shrew provides a useful alternative model for the study of influenza H1N1 virus

**DOI:** 10.1186/1743-422X-10-111

**Published:** 2013-04-10

**Authors:** Zi-feng Yang, Jin Zhao, Yu-tong Zhu, Yu-tao Wang, Rong Liu, Sui-shan Zhao, Run-feng Li, Chun-guang Yang, Ji-qiang Li, Nan-shan Zhong

**Affiliations:** 1 The First Affiliated Hospital of Guangzhou Medical University, State Key Laboratory of Respiratory Disease (Guangzhou Medical University, China), Clinical Virology Division, 1 Kangda Road, Guangzhou, 510230, China; 2 Macau University of Science and Technology, Faculty of Chinese Medicine, Macau SAR, AvenidaWai Long, Taipa, Macau, 999078, China; 3 Traditional Chinese Medicine of Guangzhou University, Centre for Artemisia apiacea, 12 Airport Road, Guangzhou, 510405, China; 4 Guangdong Provincial Hospital of Traditional Chinese Medicine, Emergency Department, 111 Dade Road, Guangzhou, 510120, China

**Keywords:** Influenza H1N1 virus, Tree shew, Clinical signs, Replication, Pathological changes, Receptors

## Abstract

**Background:**

The influenza pandemics have resulted in significant morbidity and mortality worldwide. Animal models are useful in the study of influenza virus pathogenesis. Because of various limitations in current laboratory animal models, it is essential to develop new alternative animal models for influenza virus research aimed at understanding the viral and host factors that contribute to virus infection in human.

**Method:**

We investigated the replicative efficiency of influenza H1N1 virus (classic strain (Influenza A/PR/8/34), seasonal influenza isolate (A/Guangzhou/GIRD/02/09) and swine-origin human influenza virus (A/Guangzhou/GIRD/07/09)) at Day1,2,4,6 and 9 p.i. using TCID_50_ and qPCR assay in tree shrew model. Body temperature was monitored in the morning and evening for 3 days before infection and for 14 days. Seroconversion was detected by determining the neutralizing antibody titers against the challenge viruses in the pre- and exposure serum samples collected before infection and at 14 days p.i., respectively. Lungs and tracheas of tree shews were collected at day 14 post p.i. for histopathological analysis. Lectinhistochemistry analysis was conducted to identify the distribution of SAα2,3 Gal and SAα2,6 Gal receptors in the lung and trachea.

**Results:**

The infected tree shrew displayed mild or moderate systemic and respiratory symptoms and pathological changes in respiratory tracts. The human H1N1 influenza virus may replicate in the upper respiratory tract of tree shrews. Analysis of the receptors distribution in the respiratory tract of tree shrews by lectinhistochemistry showed that sialic acid (SA)α2,6-Gal receptors were widely distributed in the trachea and nasal mucosa, whereas (SA)α2,3-Gal receptor was the main receptor in the lung tissue.

**Conclusions:**

Based on these findings, tree shrew seemed to mimic well influenza virus infection in humans. We propose that tree shrews could be a useful alternative mammalian model to study pathogenesis of influenza H1N1 virus.

## Background

Influenza viruses infecting humans cause a range of illnesses from unapparent infections to pneumonia and severe acute respiratory syndrome [1]. Recently, efforts have been increased to understand the pathogenesis of the various influenza virus infections and to develop new methods of treatment [2]. Thus, it is essential to have laboratory animal models that replicate the major features of illness in humans and provide selective and reproducible results. This selected animal model needs to mimic human influenza, in terms of similarity of clinical signs, histopathologic changes and virus replication kinetics. A number of animal models such as mice [3], cotton rats [4], guinea pigs [5], hamsters [6], ferrets [7], non-human primates, such as macaques [8] have been developed, but many gaps, including clinical symptoms and transmission, remain in our understanding.

Although various laboratory animals have been used in influenza virus study, each of them has particular advantages and disadvantages. Generally, mice, guinea pigs and hamsters models are widely used for influenza virus research. However, they do not exhibit some of the clinical symptoms detected in humans such as nasal exudates, fever, sneezing, and coughing, and only display hypothermia and weight loss. Additionally mice, unlike other rodents like guinea pigs, cotton rats and hamsters, cannot be infected with primary human virus clinical isolates readily, and thus are in mostly used in the research of mouse-adapted strains [9]. Therefore, the pathogenesis of influenza virus could not be studied adequately in rodent models as generally recognized. It is well-known that ferrets and non-human primates (e.g. macaques) are excellent mammalian animal models for studies of influenza virus pathogenicity and host immunity, and moreover, the clinical signs of influenza virus infection in ferrets resemble those in humans [10,11]. Although these species provide useful models for influenza virus pathogenesis studies, some disadvantages of those such as availability, cost, husbandry demands and ethical constraints limit the use of them for such research [12]. Until recently, more efforts have been focused on development of animal models in attempt to provide more alternative animal models for study of influenza virus pathogenesis and antivirals.

The tree shrews (*Tupaiabelangeri*, family Tupaiidae) are now widely classified as a separate taxonomic group of mammals (Scandentia) that probably diverged from the primate order (Primates) about 85 million years ago [12,13]. Consequently, tree shrews are phylogenetically much more closely related to humans, which make it a useful animal model for some human viral diseases in Southeast Asia [14]. Currently, tree shrew models are mainly used for research into the nervous, digestive and urinary systems, among others [15,16]. In the 1980s, tree shrews were already being used for animal models for Epstein–Barr virus [17] and rotavirus[18] infection. The tree shrew models have also been used widely for infection with hepatitis A virus [19], hepatitis B virus [20,21], hepatitis C virus [22], measles virus [23], adenovirus [24,25], herpes simplex virus [26], respiratory syncytial virus [27], human immunodeficiency virus [28], and rotavirus [18].

Therefore, we hypothesized that clinically apparent infections can arise from infection of influenza viruses in tree shrew, and tested our hypothesis to evaluate the tree shrew to be potential in the study of influenza. In the present study, we established a small alternative model, the tree shrew, which could be contributed to further study of human influenza virus infection.

## Results

### Virus replication in tree shrews and pathological changes

**Figure 1:**
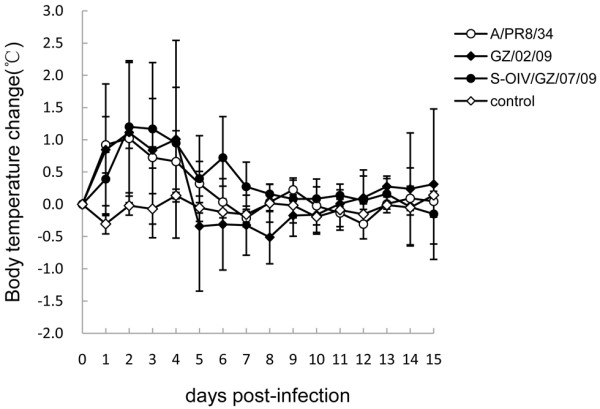
Changes in body temperature in tree shrews infected with H1N1 influenza viruses.

To evaluate virological characteristics in susceptible tree shrews, 9 tree shrews (3/group) were intranasally challenged with 10^5^ TCID_50_ (50% tissue culture infectious dose) of the H1N1 strains A/PR/8/34, GZ/02/09 and S-OIV/GZ/07/09, respectively. Control animals were inoculated with an equal volume of uninfected allantoic fluid. We examined all clinical symptoms during the course of infection to characterize the disease caused by virus infection. All tree shrews were observed to be clinically normal signs on day 0. Clinical signs of infection were initially observed on day 1 post-infection (p.i.), with most animals exhibiting signs by day 3 or 4 p.i. and gradually disappeared by day 9 p.i. Clinical signs observed in three infected groups included hypoactivity, and increasing nasopharyngeal secretion (Table 1). There was no obvious change in average body weight in animals inoculated with the viruses compared with the control group (Table 1).We observed slight fevers in all three strains of influenza viruses on day 2 and 3 p.i. (Figure 1). However, the result varied individually. Larger numbers of animals are needed to be acquired for future studies.

**Table 1:** Virus, clinical signs, virus replication, and seroconversion of tree shrews

**Virus**	**Body weight (g)**^**a**^	**Body temperature (°C)**^**b**^	**Nasopharyngeal secretion**	**Peak mean nasal wash titer±SD (day)**^**d**^	**Number positive/total (average logTCID**_**50**_**/mL)**^**e**^			**Seroconversion(HI titer)**^**f**^
					**Nasal mucosa**	**Tracheal**	**Lung**	
A/PR8/34	99.77±0.42	39.78±0.44	Positive (3/3)^**c**^	2.94±0.92 (2)	(3/3) (1.89)	(0/3)	(0/3)	(3/3) 80,80,320
GZ/02/09	98.92±0.63	40.26±0.55	Positive (3/3)	3.00±0.33 (2)	(3/3) (1.90)	(0/3)	(0/3)	(3/3) 80,160,640
S-OIV/GZ/07/09	100.24±0.49	40.57±0.59	Positive (3/3)	4.24±0.25 (1)	(3/3) (2.17)	(0/3)	(0/3)	(3/3) 80,160,640
Control	99.46±0.36	38.20±0.47	No	0	(0/3)	(0/3)	(0/3)	(0/3) 0,0,0

^a^ Bodyweight on day o.

^b^ Average body temperature on day 2 p.i.

^c^ Number of inoculated tree shrews/total number.

^d^ Peak nasal wash titers are expressed as the mean±SD log10 TCID_50_/mL.

^e^ Virus titers in different tissues was detected on day 2 p.i.

^f^ Serum was collected on day 14 p.i., and homologous strains were used with chicken RBCs in HI assay.

**Figure 2:**
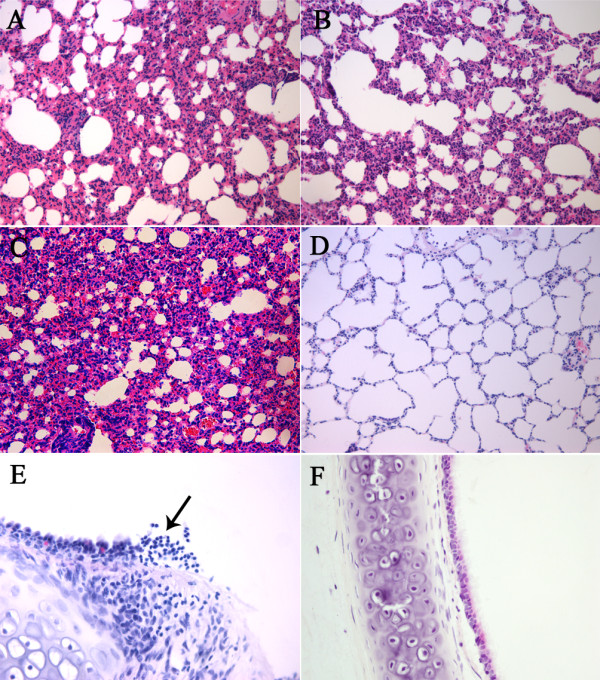
**Clinical pathology of infected tree shrew.** (**A**) lung from tree shrew challenged with A/PR8/34 virus with interstitial pneumonia, inflammatory cell infiltration, hemorrhage, edema, and lung exudate interval; (**B**) lung of tree shrew challenged with A/Guangzhou/GIRD/02/09 virus with interstitial pneumonia; (**C**) lung of tree shrew challenged with swine influenza virus A/Guangzhou/GIRD/07/09 with interstitial pneumonia, interstitial edema and hyperemia; (**D**) control lung tissue; (**E**) Trachea from tree shrew challenged with A/Guangzhou/GIRD/02/09 virus, with destruction of ciliated epithelia and inflammatory cell infiltration; (**F**) control trachea tissue. Images A-D were taken at 200× magnification, and E-F at 400 × magnification.

A spectrum of histopathological features was found in the lungs of tree shrews infected with each influenza H1N1 virus. Evidence of acute bronchopneumonia and interstitial pneumonia were observed in the lungs of all tree shrews infected with A/PR8/34 virus (Figure 2A). Groups of GZ/02/09 and S-OIV/GZ/07/09 showed evidence of interstitial pneumonia, mild bronchiolitis (Figures 2B and C). Also, extensive interstitial edema and hyperemia were characterized in all virus challenge groups (Figures 2A, B, and C). Moreover, pathological change in trachea of GZ/02/09 group exhibited infiltration of inflammatory cells (Figure 2E). In comparison, the tracheal and lung tissue from a control tree shrew had no apparent histological changes (Figure 2D and F).

**Figure 3:**
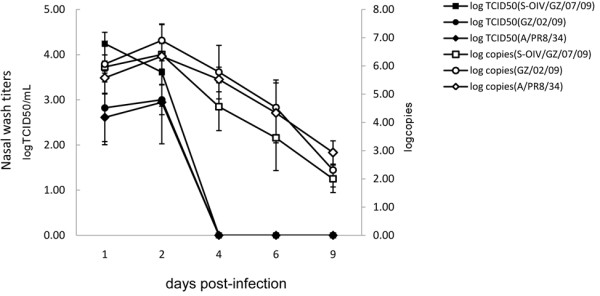
**Influenza virus kinetics of H1N1 influenza viruses infection in tree shrews.** Three groups of tree shrews (3/group) were intranasally inoculated with 10^5^ TICID_50_ of virus. At indicated time points (days 1,2,4,6 and 9 p.i.), nasal wash samples were collected. Titers (mean±SD) are presented as logTCID_50_/mL and log copies.

Nasal washes from the tree shrews were collected on days 1,2,4,6 and 9 p.i. and subjected to TCID_50_ and qPCR assays. The data showed that tested viruses grew to peak titers on day 1 or day2 p.i., with infectious virus titres ranging from 10^4.24^ to 10^2.94^ TCID_50_/ml, and dropped to undetectable levels by day 4 p.i. using TCID_50_ assay (Figure 3). This viral growth kinetics was consistent with that observed from outcome of qPCR assay for each strain tested (Figure 3). However viral shedding ended by around day 4 to 9 p.i., probably due to superior sensitivity of qPCR assay. The titer of S-OIV/GZ/07/09 was highest among all strains tested. Moreover, we detected infectious virus in homogenate of nasal mucosa with logTCID50/mL value ranging from 1.89 to 2.17, while no virus was found in trachea and lung (Table 1). Taken together, upper respiratory tract of tree shrew may be more permissive to human clinical isolates when inoculated by intranasal route.

Furthermore, tree shrews used in the present study were determined to be seronegative for influenza A viruses (H1N1), using the hemagglutination inhibition (HI) test. Some animals in each group after inoculation with the two viruses possessed recognizable HI antibody titers range from 80 to 640 Hemagglutinating Units(HAU) (Table 1).

### Distribution of avian and human influenza receptors in the respiration tracts of tree shrews

**Figure 4:**
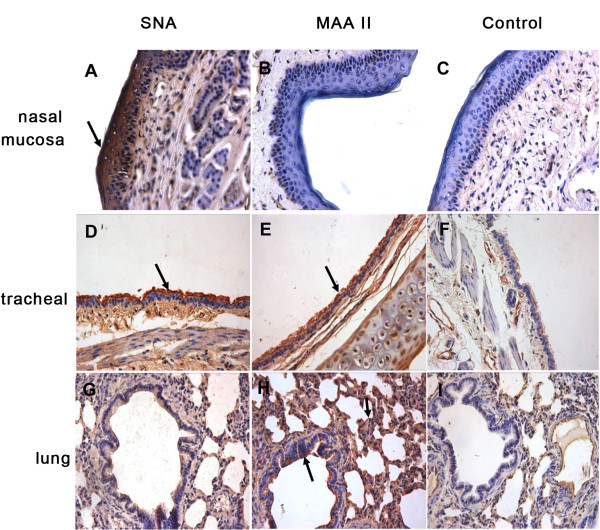
**Distribution of avian (SAα2,3) and human (SAα2,6) influenza receptors in the nasal mucosa, trachea and lung of tree shrews.** Both avian influenza virus receptor SAα2,3 Gal binding with MAA II and human influenza virus receptor SA α2,6 Gal binding with SNA are shown in brown. Stained with SNA (**A**,**D**,**G**), MAA II (**B**,**E**,**H**). SNA and MAA II lectins on sections previously treated with neuraminidase, where no faint binding was detected (**C**,**F**,**I**). A–C, Nasal mucosa. (**A**) In the nasal mucosa, stained SNA were detected on squamous epithelial cells (⬆) and vascular endothelial cells (**B**) Only a few of MAA staining was visible on squamous epithelial cells. D–F, Trachea (**D**) SNA staining was visible on almost all epithelial cells (**E**) a few stains of SA α2,3 Gal were found (⬆). G–I, lung (**G**) In the lung, non-ciliated cuboidal epithelium of the terminal bronchioles mainly expressed SAα2,6 Gal (⬆). (**H**) In the lung, alveolar epithelial cells mainly expressed SA α2,3 Gal and alveolar macrophages also expressed SA α2,3 Gal (⬆). Magnification ×400.

We detected the distribution of influenza virus receptors in a range of tissues from tree shrews. The receptor distribution was consistent within each individual animal. Using lectin staining, we found widespread distribution of both SAα2,6 Gal (*Sambucusnigra* agglutinin; SNA) and SAα2,3 Gal(*Maackiaamurensis*lectin II; MAA II) receptors in the respiratory tract. In the nasal mucosa, SA α2,6 Gal receptors were widely expressed on the squamous epithelial cells, vascular endothelial cells and the epithelial cells of the gland, only a few SAα2,3 Gal receptor were detected on squamous epithelial cells,(Figure 4A, B). The SA α2,6 Gal receptor was mostly detected in the pseudostratified ciliated cells of the trachea, whereas only a few SAα2,3 Gal were found in the same area (Figure 4D,E). In the mixed glands of the submucosa layer, both receptors in endothelial cells of blood vessels were detected (Figure 4D,E). In lung tissue, the non-ciliated cuboidal epithelium of the terminal bronchioles mainly expressed SAα2,6 Gal (Figure 4G), whereas alveolar epithelial cells mainly expressed SAα2,3 Gal, and alveolar macrophages also expressed SAα2,3 Gal (Figure 4H). Treatment with neuraminidase prior to lectin staining resulted in absence of staining and thus confirmed the specificity for both SNA and MAA II (Figure 4C,F,I). The distribution of SAα2,6 Gal was mainly detected in the trachea and bronchus and to a lesser degree in the alveolar cells. In contrast, SAα2,3Gal receptor was more regularly observed in respiratory bronchiolar and lung alveolar cells, and only sporadic expression of SAα2,3Gal was observed in the tracheal, bronchial and bronchiolar epithelial cells.

## Discussion

We used a tree shrew (*TupaiaBelangeri*, family Tupaiidae) model to study clinical signs, virus shedding, pathology of influenza virus A H1N1 and sialic acid receptor type distribution. Our results demonstrated that influenza H1N1 virus replicated efficiently in respiratory tract of tree shrews, and showed mild or moderate clinical signs and pathological changes. These findings in tree shrews seemed in accord with related manifestations in human influenza infections [1]. It also revealed that upper respiratory tract of tree shrew may be more permissive to human clinical isolates when inoculated by intranasal route. Patterns of influenza virus receptor distribution in the upper and lower respiratory tract are also similar in tree shrews and humans [29-32]. Taken together, our results suggested that tree shrews could be a promising alternative animal model for the study of influenza pathogenesis.

Main clinical signs in this model include slightly increased body temperature and nasal secretion, but anorexia and lethargy were not obvious. Although sneezing occurred occasionally, it was more frequently associated with the common cold than with influenza. Tree shrews usually had fever after inoculation with high challenge dose of influenza virus, and dropped to undetectable levels by around one week, which was similar to human infection timeline [1]. Because both of systemic and respiratory symptoms were characteristically observed in tree shrews infected, indeed the symptomatology found in tree shrews was partly similar to human influenza infections without complication [1]. Additionally, the disease manifestations of influenza virus infection in tree shrews also partly resembled those in an excellent mammalian animal model (eg. ferret) [7]. However, loss appetite, congested eyes and otologic manifestation were not observed in tree shrews, but in ferrets [33,34]. The mouse model can manifest no obvious clinical signs of influenza-like illness, but develop severe pneumonia. Thus far, the tree shrews seem not superior to the ferret model, but have the advantage over the rodent model in the clinical similarity. The serological data showed that tree shrews readily seroconvert in response to intranasal inoculation of virus, and serum neutralizing antibody titers of infected animals range from 80 to 640. Considering the clinical symptoms and antibody immune response observed in tree shrews, it is reasonable to presume this model is potential to evaluate the efficacy of antiviral agents and vaccine for the prevention of influenza infection. However, the tree shrew infected with human influenza model didn’t result in lethality, thereby detection of increasing nasal wash titers can serve as endpoints for determination of vaccine efficacy.

In particular, in our study the tested influenza H1N1 viruses (including classic strain, seasonal isolate and novel swine H1N1) could infect the tree shrews without prior adaptation. Thus, tree shrew model could be one of the attractive options for the study of pathogenesis and antiviral agent shortly once new influenza viruses emerge.

Like humans, tree shrews inoculated with human influenza viruses demonstrated a primarily upper respiratory tract infection. Influenza viruses could be isolated at high titers from nasal washes, but no virus could be detected in tracheas and lungs. In addition, the infections in tree shrew were only induced by the high virus challenge dose (up to10^5^ TCID_50_), without mortality. Nevertheless, it is likely that the tested human influenza infections are self-limited in tree shrew model like humans. Histopathological analysis indicated that clinical isolate influenza H1N1 virus infection caused exudative and interstitial pneumonia, moderate bronchitis, mild bronchiolitis, interstitial edema and inflammatory infiltrates, which showed considerable similarities to influenza virus pneumonia in human [35] and this also suggested that damage to the ciliated epithelium may be caused by the inflammatory response.

It is important to note that receptors play a crucial role in determining the host specificity and tissue tropism of virus [36]. The hemagglutinin of influenza viruses initiates infection by binding sialic acid (SA) that is bound to glycans through SA α2,3 Gal or SAα2,6 Gal linkage [37]. Therefore, the lectinhistochemistry data are important to evaluate the tree shrew as a model for influenza virus. We established that both human influenza (SA a2,6-Gal, SNA) and avian (SA a2,3-Gal, MAA II specific) receptor types were present in tree shrew respiratory tract, with each tissue showing distinctive anatomical distribution of the two receptors. This suggested that the respiratory tracts may be permissive to viral entry or infection. In tree shrews, the SA α2,6 Gal receptor that is more frequently associated with human influenza viruses was restricted primarily to the trachea and some bronchus, whereas the SAα2,3 Gal receptor preferentially bound by avian viruses was more abundantly present in the pulmonary alveoli and respiratory epithelium, which was similar to humans and ferrets [36,37]. Considering the anatomical receptor distribution in upper and lower respiratory tract in tree shrew, it is reasonable to hypothesize that the tree shrew can be infected with human influenza viruses, but also potentially be infected with high pathogenic avian influenza virus, even resulting in a lethal pneumonia model. However, this requires further investigation.

Currently, tree shrews are widely used in medical and biological research, especially in virology. However, tree shrews, used as experimental animals, need a suitable source for this study. Many countries now carry out laboratory research on tree shrews, such as in the German Primate Center [38], and tree shrews have also been bred successfully. In China, Kunming Medical University has already achieved local standards for experimental tree shrews, which provides comprehensive quality assurance for artificial propagation and experimental studies of tree shrews [39,40]. In addition, although ferret is possibly the best model for influenza in humans, the use of this species for routine research purposes is prohibitively expensive [12,37]. However, compared to ferrets, tree shrews can be bred more easily and are relatively inexpensive to maintain for study.

In conclusion, our study aimed to determine whether the tree shrew provides a useful small alternative model for the study of influenza H1N1 virus infection. The main advantages of tree shrews as an experimental model are phylogenetically close to primates [41], susceptible to influenza without prior adaptation, small size, low expense and husbandry, which make the model more accessible to the researchers. Although the tree shrews mimic well the disease in humans, the absence of severe infection and possible differences in drug pharmacokinetics in tree shrews and humans may limit the study of antiviral treatment. Further efforts are needed to determine its pathogenesis in tree shrew model such as viral replication in the extra pulmonary organs as well as its application of assessments of antiviral agents and vaccine. To this end, the tree shrew model will be useful for assessment of circulating strains that could challenge human public health.

## Methods

### Viruses and cells

Classic strain A/PR/8/34 was purchased from ATCC (American Type Culture Collection). A/Guangzhou/GIRD/02/09 (GZ/02/09) virus was obtained from unadapted H1N1 human clinical isolate before 2009 influenza pandemic. Novel swine influenza virus (A/Guangzhou/GIRD/07/09 (S-OIV/GZ/07/09), GenBank Accession No. HM014332.1) was also an unadapted H1N1 human clinical isolate. Virus stocks were produced by passage in 10-day-old embryonated chicken eggs, which was purchased from Institute of Animal Husbandry, Guangdong Academy of Agricultural Sciences. Madin–Darby canine kidney (MDCK) cells (American Type Culture Collection) were cultured in Dulbecco’s Modified Eagle’s Medium supplemented with 10% fetal bovine serum. The TCID_50_ was determined for MDCK cells after incubation at 37°C for 3 days [42], and TCID_50_ values were calculated using the Reed–Muench method [43]. All experiments were performed at biosafety level 2. All personnel were required to use respiratory protection during working with live viruses or infected animals.

### Ethics statement

All animal research was approved by the Guangdong Provincial Department of Science and Technology (approval ID, SYXK (YUE) 2008–0093), which complied with the guidelines of Guangdong Regulation for Administration of Laboratory Animals (2010), and the guidelines on the welfare of non-human primates were used in research. Animals were allowed free access to food and water and kept on a 12-h light/dark cycle, received environmental enrichment and were monitored daily for evidence of disease and changes in attitude, appetite, or behavior suggestive of illness. In cases of suffering animals were treated with anesthetic or otherwise the experiment was stopped by humane killing with subsequent post mortem analysis.

### Animals

3-month-old male tree shrews weighing 95-105 g were obtained from the Animal Experimental Centre of Kunming Medical University. They were seronegative by HA inhibition assay to influenza A/PR/8/34, A/Guangzhou/GIRD/02/09 and A/Guangzhou/GIRD/07/09 human H1N1virus isolates.

### Viral inoculation of tree shrews and viral titre quantification

Twenty four tree shrews, that were seronegative for influenza virus were used in the study. Each animal was lightly anesthetized with a solution of ketamine/xylazine/atropine, formulated to provide doses of 25 mg/kg ketamine, 1.7 mg/kg xylazine and 0.05 mg/kg of atropine to each animal. To evaluate the ability of different viral strains to replicate in tree shrews, ~10^5^ TCID_50_ was administered to each animal intranasally in each group of six tree shrews; three control animals were inoculated with an equal volume of uninfected allantoic fluid. Clinical signs of infection and bodyweight and temperature were recorded daily. In order to measure their body temperatures accurately, animals were quiet after 10 min when captured. To detect viral titer in respiratory tracts, three animals in each group were euthanized at day 2 p.i.. Samples of nasal mucosa, trachea and all lobes of the lung were collected and stored at −80°C. The tissues were weighed and subsequently homogenized to make a 10% weight by volume (w/v) suspension for virus titration on MDCK cells using TCID_50_ assay. Nasal wash samples containing 0.3 mL PBS with antibiotic were collected from all tree shrews on days 1, 2, 4, 6 and 9 for viral titer determination using the TCID_50_ assay. Also, we performed quantitative PCR (qPCR) detection of virus in nasal wash samples by use of an Applied Biosystems Prism 7500 system,as described [44]. Nasal wash samples were made into aliquots and placed on dry ice immediately after collection. Samples were stored at −80°C until use. Representative sections of the left lung and trachea were collected from each euthanized or deceased animal for viral load detection. Samples were snap frozen in liquid nitrogen and stored at −80°C until analysis.

### Serological assays

Serum samples obtained from tree shrews before exposure were assayed in an HI assay with inactivated antigens for challenge human influenza A viruses. Seroconversion was detected by determining the neutralizing antibody titers against the challenge viruses in the pre- and post-exposure serum samples collected before infection and at 14 days p.i., respectively, by means of virus neutralization in MDCK cells, as described previously [45].

### Histopathological analysis

Lungs and tracheas of euthanized tree shrews were preserved in 10% phosphate-buffered formalin. Tissues were then processed for paraffin embedding and cut into 4-μm-thick sections. One section from each tissue sample was subjected to standard hematoxylin and eosin staining, while another was processed for lectinhistochemistry.

### Lectinhistochemistry

Two tree shrews were used for sample collection for characterization of sialic acid receptor type. Expression of SAα2,3 Gal and SAα2,6 Gal receptors in the lung and trachea was measured using lectinhistochemistry. Before histochemistry, neuraminidase pretreatment was performed to confirm the specificity of the lectin stains. All paraffin-embedded consecutive sections of 4-μm thickness from all of these organs, which were four serial sections, were stained with biotinylated, SA-specific lectins. Paraffin-embedded tissue sections were deparaffinized and immersed in 0.3% hydrogen peroxide methanol to eliminate endogenous peroxidase activity. Then, each slide of tissue sections was covered with 10 U/μl neuraminidase (NEB, Ipswich, MA, USA) for 24 h incubation at 37°C. Additional negative controls were performed by using slides incubated with PBS. Biotinylated MAA II, which is specific for α-2,3-linked SA (a marker for avian influenza virus receptor), was purchased from Vector Laboratories (Burlingame, CA, USA), and biotinylated SNA, specific for binding the α-2,6 linked sialic acid (a marker for human influenza virus receptor), were purchased from Vector Laboratories. Lectinhistochemistry was performed as described previously [30]. Briefly, the formalin-fixed, paraffin-embedded tissue sections were deparaffinized and 5% bovine serum albumin was used to block non-specific staining. Sections were pre-soaked in PBS and blocked using a biotin–streptavidin blocking kit (Vector Laboratories) according to the manufacturer’s instructions. The tissue sections were incubated with SNA (0.625 μg/ml) and MAA (1.25 μg/ml) in buffer at 4°C for 1 h. Then, all tissue sections were incubated in ready-to-use horseradish peroxidase streptavidin (Vector Laboratories) buffer for 30 min. Biotinylatedlectin binding was visualized using a DAB substrate chromogene kit (Maixin, Fuzhou, China), which gives a brown color, and slides were counterstained with hematoxylin. Negative controls were slides incubated with PBS instead of the lectin.

## Abbreviations

p.i.: post-infection.

## Competing interests

The authors have no conflicts of interest.

## Authors’ contributions

NSZ, ZFY, JZ, YTZ, YTW, RL and JQL co-conceived this study. JZ performed histopathological and lectinhistochemistryanalysis. RL and CGY were responsible for the animal experiments. YTZ, YTW, SSZ and RFL were responsible for virus titration and serological analysis. ZFY, JZ and RFL contributed to writing the paper, NSZ and ZFY examined and approved the final manuscript. All authors read and approved the final manuscript.
